# Environmentally Related Diseases and the Possibility of Valuation of Their Social Costs

**DOI:** 10.1155/2014/284072

**Published:** 2014-10-14

**Authors:** Ilona Hajok, Ewa Marchwińska, Grzegorz Dziubanek, Bernadeta Kuraszewska, Agata Piekut

**Affiliations:** Department of Environmental Health, Faculty of Public Health, Medical University of Silesia in Katowice, Ulica Piekarska 18, 41-902 Bytom, Poland

## Abstract

The risks of the morbidity of the asbestos-related lung cancer was estimated in the general population of Poles as the result of increased exposure to asbestos fibers during the removal of asbestos-cement products and the possibility of the valuation of the social costs related to this risk. The prediction of the new incidences was made using linear regression model. The forecast shows that to the end of 2030 about 3,500 new cases of lung cancer can be expected as a result of occupational exposure to asbestos in the past which makes together with paraoccupational exposure about 14.000 new cases. The forecast shows the increasing number of asbestos-related lung cancer in Poland and indicates the priority areas where preventive action should be implemented.

## 1. Introduction

More than 30 years of research in the environmental health field confirm an important role of chemical compounds present in the environment in the etiology of many diseases. The number of scientific reports on the increasing health risks from long term exposure to chemicals present in our daily lives is rapidly rising. The results of the research allow grouping the chronic diseases, whose prevalence is variably dependent on the exposure of the population to chemical pollutants. The five categories [1] are

- certain types of cancer [2, 3],
- developmental disabilities, including learning disabilities [3–6],
- Alzheimer's and Parkinson's diseases [7–11],
- reproductive health and fertility problems [12],
- asthma [3].

The costs borne by society as a result of chronic diseases are enormous. It is estimated that the financial burden for the EU healthcare systems in 2006 associated only with a group of cardiovascular diseases was slightly less than 110 billion euros. This gives the annual cost of 223 euros per capita or about 10 percent of the total health care spending in the EU. The group of asbestos-related diseases, including lung cancer, resulting from the exposure to asbestos present in the environment are the best proven example of close association between environmental pollution and some diseases.

Research has shown that exposure to asbestos is not limited only to occupational exposure and even though in majority of developed countries the use of asbestos for the manufacture of various products was discontinued many years ago, the number of asbestos-related diseases is increasing [13]. Taking into consideration the fact that the average latency period of asbestos-related diseases is 30 years, the effects of this exposure will be even more noticeable and may affect up to 40% of adult men in Poland [14, 15]. According to the report of US EWG (Environmental Working Group) asbestos-related disease is responsible for the death of 1 out of every125 American men over the age of 50 [16].

Each year in the USA there are approximately 10,000 deaths only due to diseases resulting from exposure to asbestos fibers. It is estimated that in the next 40 years asbestos will be the cause of 100,000 deaths of Americans [16]. Exposure to asbestos fibers may represent multiple risks including lung cancer, interstitial fibrosis of lung tissue (asbestosis), and pleural fibrosis or pleural mesothelioma [17]. The lung cancer, pleural mesothelioma, and peritoneal disease concern not only workers of asbestos mining or processing plants but also users of asbestos products [18]. Each year of usage and disposal of asbestos-cement products the environmental exposure to asbestos fibers will be increasing. In recent years the problem of asbestos fibers emission has grown rapidly in Poland, due to the fact that the durability of asbestos-cement panels is estimated at about 30 years [19]. This means that the life time of asbestos products used in the 60s, 70s, and 80s of the 20th century is coming to an end. Furthermore, the national program adopted by the Polish Government in 2002, “Elimination of asbestos and asbestos contained products used in Poland”, is to remove all asbestos-containing products from the country by the end of 2032 [20].

Paradoxically, the environmental risk associated with asbestos is increasing as the effect of individual removal of degraded asbestos panels by private persons, as well as violations of the health and safety regulations by the companies specialized in asbestos removal and illegal disposal of asbestos waste, is rampant.

The estimation of the risks of asbestos-related lung cancer in the general population, under the increase of exposure to asbestos fibers resulting from the removal and disposal of asbestos products in the country, as well as exposure from other activities such as drilling or sawing asbestos-cement sheeting, is a crucial problem requiring preventive actions.

The principal aim of this paper is to estimate the risks of the morbidity of the asbestos-related lung cancer in the general population of Poles as the result of increased exposure to asbestos fibers which occurs during the removal and disposal of asbestos-cement products in Poland. The second aim was to examine the possibility of valuation of social costs related to these risks. Asbestos-related lung cancer, with a median latency period of about 30 years [21, 22], stands out among other asbestos-related diseases because it occurs mainly in the case of the elderly. Additionally, in the case of people suffering from asbestosis, the risk of developing this cancer is increased up to five times [13].

## 2. Methods

The data used in the study was derived from databases of the authorities and the national institutions (i.e., the Ministry of Economy, Municipal Offices, Central Statistical Office, National Institute of Public Health-PZH, the Institute of Occupational Medicine and Environmental Health in Sosnowiec, and the data coming from AMIANTUS PROJECT, prophylactic examinations of the former workers of the asbestos processing plants).

In order to prepare the prediction, the number of the population occupationally and paraoccupationally exposed to asbestos fibers in Poland was estimated. The occupationally exposed population was estimated on the basis of the data on employees in 28 asbestos-processing plants in the country, that is, 58,000 people [23]. Size of the population exposed paraoccupationally to asbestos was estimated at 230,000 people, based on the data from the questionnaire used among workers, which shows that the average number of the worker's family consist of four people.

According to estimates of the Ministry of Economy, in Polish territory there is a total of 15 466 000 tonnes of asbestos products, which corresponds to 1 351 500 000 m^2^. The most widespread use of asbestos-cement panels took place in rural areas, especially in the regions of Central and Eastern Poland. An inventory carried out in 2004–2006, which covered only 41% of all asbestos products from all the country, showed that currently 80% of asbestos-cement roofing materials need to be removed because of their poor condition, which represents a threat to the health of the users [24].

Taking into consideration the concentration of asbestos fibers in the air of particular administrative area and the number and type of former plants producing asbestos-cement products, as well as standardized incidence rates (SIR) of asbestos-dependent diseases in the population above 17 years old, three zones of the country with varying degrees of risk of asbestos-related disease were isolated [25].

In order to predict the incidence of asbestos-related lung cancer, the Poisson log-linear regression model was used [26]. It seems to be the most appropriate for count data, particularly when counts observed are close to zero. A log-linear Poisson regression results in a model with exponential growth of the number of cases because the data implies an increasing rate of incidence. The estimate covers the period ending in 2030 because by the year 2030 Polish cleansing program with asbestos must be implemented.

The possibility of evaluating the social costs associated with the risk of asbestos-related lung cancer in the general population of Poles was considered on the basis of the available literature.

## 3. Results 

The largest concentration of asbestos waste is in Malopolskie and Lubelskie administrative areas and this area has been marked in the forecast as the zone III. In the zone III, with the total area of only 13% of the whole country, there were about 50% of all registered cases of diseases resulting from exposure to asbestos dust in Poland. In this area, five large processing plants of asbestos existed, and the average concentration of airborne fibers close to a source of emission was the highest identified in the country, above 1,000 fibers/m^3^ [27].

**Figure 1:**
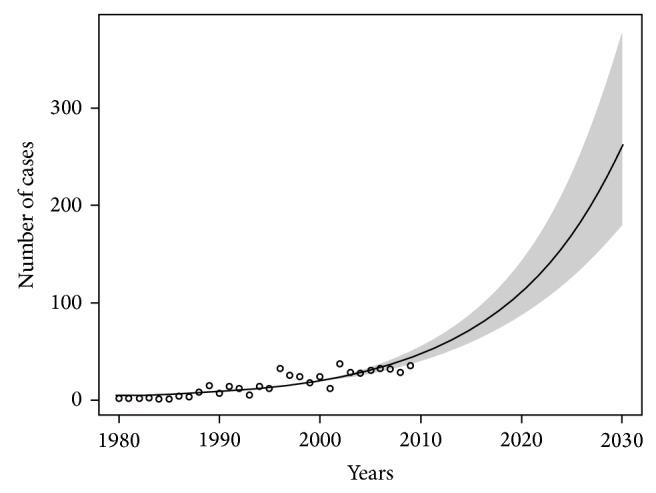
The forecast of increasing incidence of asbestos-related lung cancer in Poland until 2030. Significance level of model: *P* < 0.01.

The Lodzkie, Dolnoslaskie, Slaskie, and Mazowieckie administrative areas are in the zone II, and the rest of the regions belong to the zone I, where the amount of asbestos products is the smallest. On the basis of the prediction model, calculated for asbestos-related lung cancer, it was shown that the maximum number of new cases in Poland by the end of 2030 can be estimated at 3,500 cases. This means that the neoplastic effects of occupational and paraoccupational exposure to asbestos can cause 14,000 new cases of asbestos-related lung cancer (Figure 1). The estimate is based on the data from the questionnaire used among workers, which shows that the average worker's family consists of four people.

**Figure 2:**
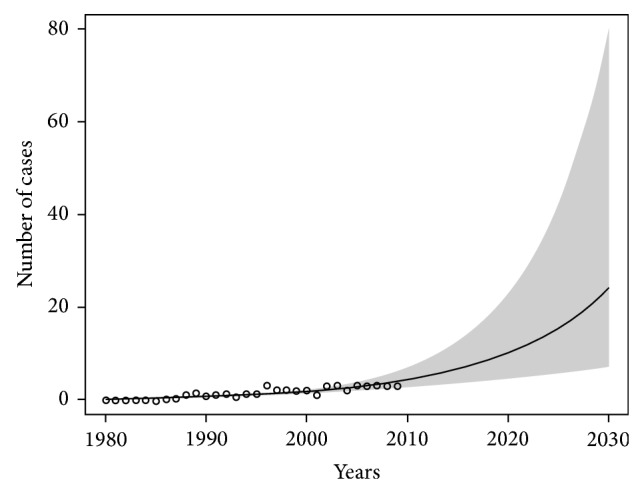
The forecast of increasing incidence of asbestos-related lung cancer in the zone number I until 2030. Significance level of model: *P* < 0.01.

**Figure 3:**
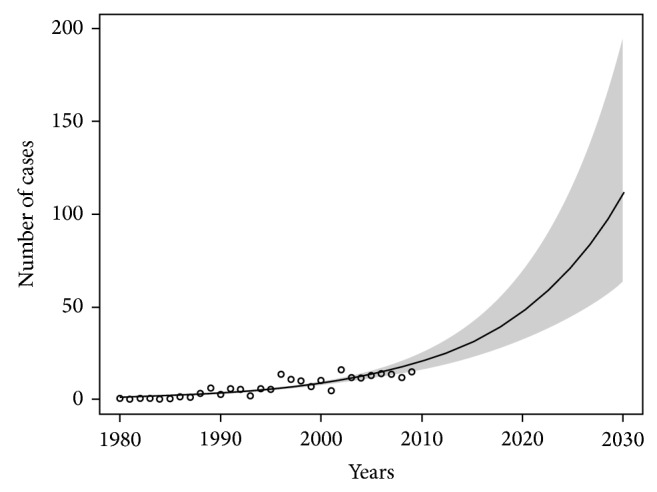
The forecast of increasing incidence of asbestos-related lung cancer in the zone number II until 2030. Significance level of model: *P* < 0.01.

**Figure 4:**
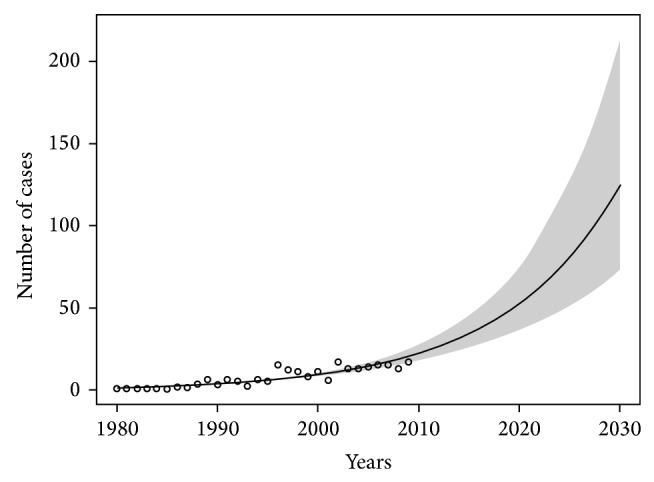
The forecast of increasing incidence of asbestos-related lung cancer in the zone number III until 2030. Significance level of model: *P* < 0.01.

Prediction models of the asbestos-related lung cancer in Poland and in each of the three zones of the country have been presented in Figures 2, 3, and 4. The smallest increase in the number of new cases of asbestos-related lung cancer was expected in the zone I where the estimated maximum number of new cases (in the years 2010–2030) may be about 640. In the zone II, we can expect about 1,800 and in the zone III are 1,900 new cases of the disease.

For estimation of the number of population occupationally and paraoccuppationally exposed to asbestos fiber in Poland, the data collected by the Ministry of Economy were used. They calculated the total number of people employed in 28 plants which used asbestos in manufacturing and are listed in the Annex 4 to the Act of 19 June 1997 on the ban on the use of products containing asbestos. On that basis, it was estimated that in Poland in the years 1940–1998 about 58 000 people were occupationally exposed to asbestos fibers. Taking into consideration the number of workers in other sectors of the Polish economy, such as building industry and shipbuilding industry, the number of people who had occupational contact with asbestos fibers can reach up to 150 000, 30% of which were exposed to asbestos in excess of the maximum allowable concentration [14].

Assuming that the families of the asbestos industry workers have also been exposed to asbestos fibers, in slightly lesser extent than the workers, mainly due to the widespread access to asbestos products and asbestos wastes, as well as because of asbestos waste used at the place of living, it was estimated that the number of the population occupationally and paraoccupationally exposed to asbestos in Poland, may reach 230 000 people, which is about 0.6% of the total population.

The calculated exposed population to asbestos fibers seems to be underestimated, because many factors may affect the total number of people exposed paraoccupationally, for example, multigenerational families, making available asbestos wastes to the neighbors, and so forth.

## 4. Discussion

According to the prediction model developed for asbestos-related lung cancer, to the end of 2030 in Poland, we can expect maximally about 14,000 new cases resulting from occupational and paraoccupational exposure to asbestos in the past. Taking into consideration the environmental exposure to asbestos, associated with aging of cement-asbestos panels on the buildings and individual removal of asbestos by some inhabitants of the country, we can expect that the number of cases of asbestos-related lung cancer will be much greater. In Western Europe, half a million deaths only due to asbestos-related mesothelioma are expected over the next 25 years [28]. These results indicate a huge burden of cost of chronic diseases to society, both in Poland and in other countries in the world. In Poland these costs are difficult to estimate. For some time, the attention of demographers, epidemiologists, economists, social politicians, and economists has been concentrated on the problem of increased expenditures for health care. Even the most developed and richest countries attach great importance to the rational spending on health care. The constant increase of the cost in healthcare is the reason of rising interests in the evaluation of their components. In the context of the whole society, health can be valued by losses incurred by the economy, such as absence from work, disability, inability to work, and death, as well as the expenses of the state budget for the treatment of diseases and for health promotion. Descriptions of these losses require monetary valuation. Good functioning of the tools, which allow efficient use of financial resources for health care, should be a priority for health policy.

By the development of electronic reporting systems and the involvement of many people, it is possible to calculate the actual costs borne by the National Health Fund (NHF) related to lung cancer. According to the NHF studies, the financial burden of cancer care of patients with lung cancer has increased twice in eight years [29]. Rising costs are associated with the increasing number of patients and with the introduction of newer and more expensive drugs and medical technology. Considering the character of the disease, in particular the lack of effective prevention, it can be concluded that this trend will continue in the next years. It should also be taken into consideration that the indirect costs of the disease may be significantly higher than the direct costs, which are associated only with the diagnostics and therapy of cancer. The knowledge and correct classification and calculation of costs of treatment are essential in order to permit proper planning and analysis regarding the financial burden on health care, as well as to calculate their impact on the economy. This is accompanied by the development of research on the economic and social burden of disease and disability. Therefore, considering the disease as a medical incident, and as an economic issue, is particularly important to estimate the full economic effects that it brings. In developed countries this kind information is available, whereas in Poland there are no reliable estimates of the total economic burden of disease and the costs of disability. The financial burden of the disease should be evaluated not only as expenditures for health services, but also as the costs for the whole national economy. The costs in the economy can be divided in various ways. In the economic valuation of diseases, a simple division into three main groups of costs is usually used [30]:

- direct costs (which are divided into medical and nonmedical),
- indirect costs (some authors divide into nonmedical and medical),
- immeasurable costs, also known as intangible.

Other classifications used less frequent in pharmacoeconomics, but very popular in the economy, takes into account two types of the costs: fixed and variable.

Fixed costs do not depend on the scale of provided services. The variable costs are related to the number of provided services. The sum of fixed and variable costs gives the total cost. The other types of costs are alternative costs which correspond to the most valuable worth of the unused alternative. Another cost is average cost which is the total cost divided by the number of units (services, goods). Then marginal cost which corresponds to the rising of the cost when the number of provided services increases by one unit. A comprehensive cost analysis permits an understanding of the economic costs of the disease which should be assessed as a whole, both as expenditure on health services and as costs incurred by the economy [31, 32].

Currently, there are few costs analyses of diseases in Poland but mainly direct costs of medical treatment of diseases are taken into consideration. The existing pharmacoeconomic analysis of diseases are related to lung and breast cancer, lymphoma, and leukemia [32, 33]. Lung cancer is one of the most common cancers, and 80% of patients have non-small-cell lung carcinoma (NSCLC).The analysis of the economy of NSCLC treatment was carried out in Germany, the USA, and Switzerland. The overall prognosis for lung cancer is poor, with five-year survival rate among less than 10% of patients.

The measure of the effectiveness of treatment routinely is tumor response but the therapy only prolongs one's life no more than a few months. Therefore, the valuation is mainly concerned with such effects as reduction of symptoms and improvement of the quality of life. Cost-effectiveness analysis showed no statistically significant differences between the therapeutic regimens used. Alternative cost analysis showed that the use of other regimens than gemcitabine (G + C) resulted in consumption of additional funds from the budget, although the direct costs of treatment regimen of gemcitabine were slightly higher [34].

The US National Cancer Institute has calculated that the burden of cancer in American society has increased from 71.5 billion dollars in 1985 to 104 billion in 1990 year. The majority of costs (55%) have been associated with premature death, 34% with the direct cost of treatment, and 11% with the working days lost. Each type of cancer has its own profile of consumption of financial outlays, which is related to the length of life of the patient, stage of disease at diagnosis, and treatment. Lung cancer is the most frequently occurring cancer in Poland. It is also the most common cause of death from all cancers among women and men. Nevertheless, in the economic analysis published mainly by the National Health Fund reporting data and medical costs incurred by the NHF are available. The selected data from the systems of Sickness Funds and NHF on costs related only to the treatment of malignant neoplasms of the bronchus and lungs were used (identified with codes C34–C34.9 according to the International Statistical Classification ICD-10). From these data, the information on asbestos-related lung cancer (C45.0) was excluded. Even the incomplete information shows that the number of patients diagnosed with lung cancer is constantly increasing. In the years 2002–2010 a steady increase of treatment costs was also observed. The cost of health care per patient was increased by about 50%. The reports of National Health Fund should be the basis for a broader discussion about the funding for lung cancer treatment in Poland as well as about the costs borne by the public payer and also about the current burden to national economy of lung cancer treatment. The direct costs of asbestos-related lung cancer which are expenditure for health services (including medications) and indirect costs which consist primarily of costs of social security (the pensions, sickness benefits) have not yet been estimated. Due to the increasing number of asbestos-related diseases, including asbestos-related lung cancer, these costs will represent a significant public health problem.

## 5. Conclusions

1. According to the prepared forecast, asbestos-related lung cancer, which is one of environmentally related diseases, will be a more and more important issue of public health in Poland, because of the increasing number of cases.
2. Applied division of the country into three zones of different, predicted occurrence of asbestos-related diseases, indicates the priority areas of prevention, where administrative and legal actions which provide reduction of public exposure to asbestos fibers should be used.
3. The predicted degree of morbidity indicates a huge burden to the public costs of chronic diseases, the rate of which in Poland is difficult to estimate with precision.
4. The development of methods of valuation of the costs of chronic diseases (in order to rationally manage financial resources) is necessary and important for effective health policy-making.
